# Can resistance training improve throwing performance in handball players? A Systematic review and meta-analysis

**DOI:** 10.1186/s13102-024-00872-y

**Published:** 2024-04-16

**Authors:** Stelios Hadjisavvas, Michalis A. Efstathiou, Irene-Chrysovalanto Themistocleous, Katerina Daskalaki, Paraskevi Malliou, Jeremy Lewis, Manos Stefanakis

**Affiliations:** 1 Department of Life Sciences, University of Nicosia, Nicosia, Cyprus; 2 Therapy Department, Central London Community Healthcare National Health Service Trust, Finchley Memorial Hospital, London, N12 0JE UK; 3 Department of Physical Education and Sport Science, Democritus University of Thrace, Komotini, Greece; 4 Professor of Musculoskeletal Research, School of Health Sciences, University of Nottingham, Nottingham, UK

**Keywords:** Resistance training, Strength training, Throwing performance, Throwing velocity, Handball players

## Abstract

**Background:**

Throwing is one of the most important movement in handball. Throwing performance is crucial for success in handball.

**Objective:**

Τo investigate the level of evidence for the effect of resistance training (RT) on throwing performance in handball players.

**Methods:**

Systematic searches of Pubmed, Medline complete, Cinahl, Sport Discus and Scopus were undertaken for peer reviewed articles published between 18 March 1995 to 18 March 2023. Randomized, controlled, clinical studies, written in English, aiming to investigate the effect at least one modality of RT on throwing performance (velocity or/and accuracy) in handball players were considered for inclusion in the study. The eligible studies were assessed for methodological quality using the Physical Therapy Evidence Database (PEDRO) scale. The Best Evidence Synthesis (BES) approach was used for synthesizing and reporting the results. Furthermore, the random-effects model was used for the meta-analysis and the Q-statistic was used to test the null hypothesis that all studies in the analysis share a common effect size.

**Results:**

One hundred ninety-eight studies were identified, of which 30 were included. A total of 727 handball players (males = 388, females = 292) were included. 28 of the 30 studies were rated as high methodological quality studies (PEDRO score > 70%) while the rest of the studies were rated as moderate methodological quality studies (PEDRO score ≤ 60%). The mean effect size for the effectiveness of resistance training (RT) in improving jumping throw, running throw, and standing throw velocity were 1.128 (95% CI 0.457 – 1.798), 1.756 (95% CI 1.111 – 2.400), and 1.098 (95% CI 0.689 – 1.507) correspondingly. Traditional weight training using barbells in mostly compound lifts yielded the most significant and robust results. Other RT modalities such as elastic bands, medicine balls, core training and ballistic training showed no significant results or positive effects due to the limited number of the studies.

**Conclusion:**

Strong evidence exists only for the effectiveness of RT using barbells in increasing throwing velocity. In contrast, the remaining RT modalities, while yielding positive results, have limited support due to limited number of studies and the high heterogeneity between studies. Furthermore, there is insufficient evidence to support various forms of RT in increasing throw distance. Finally, medicine ball training and elastic band training demonstrated no benefits in improving throwing accuracy.

**Trial registration:**

PROSPERO ID: CRD42023393574.

**Supplementary Information:**

The online version contains supplementary material available at 10.1186/s13102-024-00872-y.

## Background

Handball is an Olympic sport involving dynamic movements such as running, jumping, blocking, and throwing [1]. Throwing performance is crucial for handball success [2, 3]. A successful throw must be fast and accurate enough to ensure that the goalkeeper has insufficient time to react to block the throw [4–6]. According to Zapartidis et al. [7], throwing performance is dependent on maintaining speed and accuracy during competition. The effect of fatigue, as well as the throwing load, may be mitigated during the game, if a regular technical training program combined with RT is implemented [1]. RT is a specialized form of physical conditioning that includes gradually increasing resistive loads, various movement speeds, and different training methods such as weight machines, free weights, elastic bands, medicine balls, and plyometrics [8]. RT may increase muscle power and is utilized on a regular basis throughout the competition season [9].

Studies have investigated the impact of different RT modalities on throwing performance in handball players, such as weight training using barbells [10–14] core stability exercises [15–17], elastic band training [18, 19], plyometrics [20, 21], weight machine training [22, 23] etc. However, there is no consensus on which is the best RT modality and protocol of training to improve throwing performance. Bragazzi et al. [24] systematically reviewed the literature to answer this question in studies published before 2015. The researchers determined that RT has a notable effect on handball players by improving maximum strength, muscle power, and throwing velocity. Multiple studies have since been published [11, 14, 16, 19, 23, 25–32]. Another systematic review from Garcia et al. [33] included studies with various overhead athletes (baseball, volleyball, tennis, softball, cricket, water polo, and handball) and they concluded that specific RT for the enhancement of throwing velocity has a significant effect in all populations (male teenagers, male adults, female adults). In addition, a recent systematic review [34], concluded that RT is the most effective strategy for improving throwing velocity in elite handball players. Ηowever, this study did not investigated the influence of RT on throwing velocity in male and female non-elite handball players as well as elite females. Ιn contrast to previews systematic reviews, the present study examined the impact of various modalities of RT in both elite and non-elite male and female handball players as well as the throwing accuracy and the throwing distance.

The primary aim of this study was to investigate the level of evidence for the effect of RT on throwing performance (velocity, accuracy, distance) in handball players. The secondary aim is to propose training recommendations pertaining to the improvement of throwing performance.

## Μethods

The Preffered Reporting Items for Systematic reviews and Meta-analysis (PRISMA) statement was used [35]. This statement is intended for systematic reviews of studies assessing the effectiveness of health interventions, regardless of the study type and includes seven sections (title, abstract, introduction, methods, results, discussion, other information) with 27-item checklist [35]. A research protocol was registered in prospero database with registry code (CRD42023393574).

### Literature search

The search was carried out through the following electronic databases: Medline complete, Pubmed, Sport Discus, Cinahl and Scopus. The studies included in this systematic review were published between 1995 and 2023. In order to include studies with contemporary publication standards and adequate external validity relevant to the modern handball the year 1995 was chosen as the oldest period. The following keywords were used in the same way in each database: “resistance training” or “strengthening program” or “weight-lifting exercise programs” or “weight-lifting strengthening program” or “strength train*” or “resistance conditioning” or “weight* train*” and “throw* performance” or “throw* velocity” or “throw* accuracy” or “throw* speed” and “handball” or “handball players” or “handball athletes”. In order to screen, select and remove any duplicate article the reference manager (RefWorks, Proquest LLC) was used. Two researchers (SH and MS) performed the search independently. An additional screening of all the included studies was performed in order to identify any other suitable studies. Studies in languages other than English were excluded. Furthermore, a search was performed in grey literature using the following databases: “OpenGrey.eu”, “Clinical Trials.gov”, “WHO International Clinical Trials Registry Platform” and “Australian New Zealand Clinical Trials Registry”..

### Eligibility criteria

Inclusion criteria were established according to PICOS strategy. The “Population” (P): Handball players of all sexes and ages with no medical restrictions and free from any musculoskeletal upper extremity pain or injury before being enrolled in the study; “Intervention” (I): RT including various forms of training modalities (free weights, weight machines, plyometrics, elastic band, core stability etc.) in combination with handball training routine and with minimum duration of 4 weeks; “Comparator” (C): traditional handball training program or other RT modality in combination with handball training routine; “Outcome measures” (O): Throwing velocity, throwing accuracy, distance in the medicine ball throw; and the “Studies”: Randomized controlled trials, randomized clinical trials and crossover randomized trials. The exclusion criteria were as following: Sports athletes other than handball players, handball athletes exposed to training other than resistance training, the effect of resistance training on other variables, expert opinion, comment/commentary, editorial/letter to editor and review.

### Quality assessment

The quality of the included studies was assessed by two reviewers (SH and ICT) following the recommendations of the Physical Therapy Evidence Database (PEDRo) scale. The application of PEDRo scale in systematic reviews has demonstrated to have a fair to good reliability [36]. The scale consists of 11 criteria, of which 10 were scored. Based on the total score of the included studies, a score from 7 to 10 is considered to have high methodological quality. In contrast, a total score below 7 it is considered to have a low methodological quality. In addition, the total scores were presented as a percentage. Any disagreements regarding the methodological quality between the two assessors (SH and ICT) were first discussed and in case of no agreement, a third assessor (ME) decided for the final score.

### Data extraction and analyses

Two reviewers independently assessed the titles and/or abstracts of studies obtained from the search strategy and from additional sources. In addition the same two reviewers independently assessed the full text of potentially eligible studies. Any case of disagreement was resolved through discussion.

The assessment of the risk of bias and evidence synthesis were performed using a standardized form to extract data from the included studies. The form was custom-made in Microsoft Excel ™ in advance of data extraction. The PICO framework was used (e.g. study population and participant demographics, baseline characteristics; details of the exposure and control conditions) in order to decide which studies to include in the review.

Two reviewers (SH and MS) extracted the data independently, and discrepancies were identified and resolved through discussion. In five studies, mean and standard deviation were not presented in tables, but in graphs and attempts were made to contact the authors to obtain the data. The study authors were requested to reply within three weeks. Some informed the authors that the data were no longer available and some did not reply. Data from these five studies [16, 21, 22, 37, 38] were extracted using PlotDigitizer (V3.1.5, 2023, https://plotdigitizer.com). Three trials, at three different time points, were used until the difference between the extracted data differed only in the second decimal point between two consecutive times.

The random-effects model was used for the meta-analysis [39]. The studies included in the analysis were assumed to be a random sample from all the potential studies in this subject, and random effects analysis allows for inferences on these studies (7–9,19,31).

The main outcome measures used in the included studies were throwing velocity, throwing distance, and throwing accuracy/success. A meta-analysis was possible for throwing velocity only. This is because the majority of the studies included throwing velocity as an outcome measure, in contrast to the other outcome measures (throwing distance, and throwing accuracy/success) which vary and was not possible to run an analysis.

The throwing techniques that were mostly used were running throw, jumping throw, and standing throw. Each throw was evaluated separately, comparing each RT group (resistance training replacing some technical/tactical training) to the designated ‘control’ training. This provided higher clarity of the results and reduced the heterogeneity among trials. The assessment of throwing accuracy and goal success was evaluated by only one study. Throwing distance was assessed in five studies. One study was excluded because it did not present standard deviations (authors contacted but did not reply). One study did not have a control group (no intervention) and the other three studies each used a different throw to assess the distance. For these reasons a meta-analysis was done only for throwing velocity. Because the studies used different scales to measure the velocity, the most suitable effect size index was the standardized difference in means (d). The z test was used to test the null hypothesis that the mean effect size is zero.

#### Heterogeneity statistics

The null hypothesis, that every study in the analysis had the same effect size, was tested using the Q-statistic. The predicted value of Q would be equal to the degrees of freedom (the total number of studies minus 1) if every study had the same true effect size. To determine how much of the variety in observed effects is due to sampling error rather than variance in genuine effects, the I-squared statistic was utilised. In 95% of all similar studies/populations, the prediction interval was used as a measure of the genuine effect size [40–46]. Therefore, the following interpretations of the mean effect size are possible when comparing it with the prediction interval. The effect size can be useful in three different ways: (a) it is always helpful but ranges from a trivial to a moderate effect (both prediction interval and estimated effect size are in the same direction but the the range of the prediction interval is moderately higher); (b) it is always helpful but varies from a moderate to a big effect (range of prediction interval is substantially higher than the estimate); or (c) it may be helpful in some situations but misleading in others (prediction interval crosses zero while estimate does not crosses zero). Estimate was used as a summary of the current evidence. However, prediction interval was used to make recommendations for practitioner/clinicians as this is more representative of the true effect [47, 48]. Comprehensive Meta-Analysis Version 4 was used for the computations [41, 43, 49–51].

#### Sensitivity analysis

Sensitivity analysis was done by removing one study at the time and comparing the updated mean effect size to that of the original analysis with all the studies included using the z -test. In addition, a meta-regression using the methodological quality score as an integer variable was done.

#### Subgroup analysis

The modes of RT used in the studies were grouped into the following distinct groups: weight lifting (Barbell training), core training, elastic resistance training, medicine ball throw/training and other if it was inappropriate for any of the other categories. Subgroup analysis was done based on these groups for each of the three throws.

### Strategy for data synthesis

The best evidence synthesis (BES) was used for a qualitative assessment and to formulate conclusions. The same methodology was used by others systematic reviews [52–54]. The BES consists of 5 levels of scientific evidence and consistency was defined a priori as over 75% of studies agreeing on the same direction of results.

## Results

### Search results and selection

**Fig. 1:**
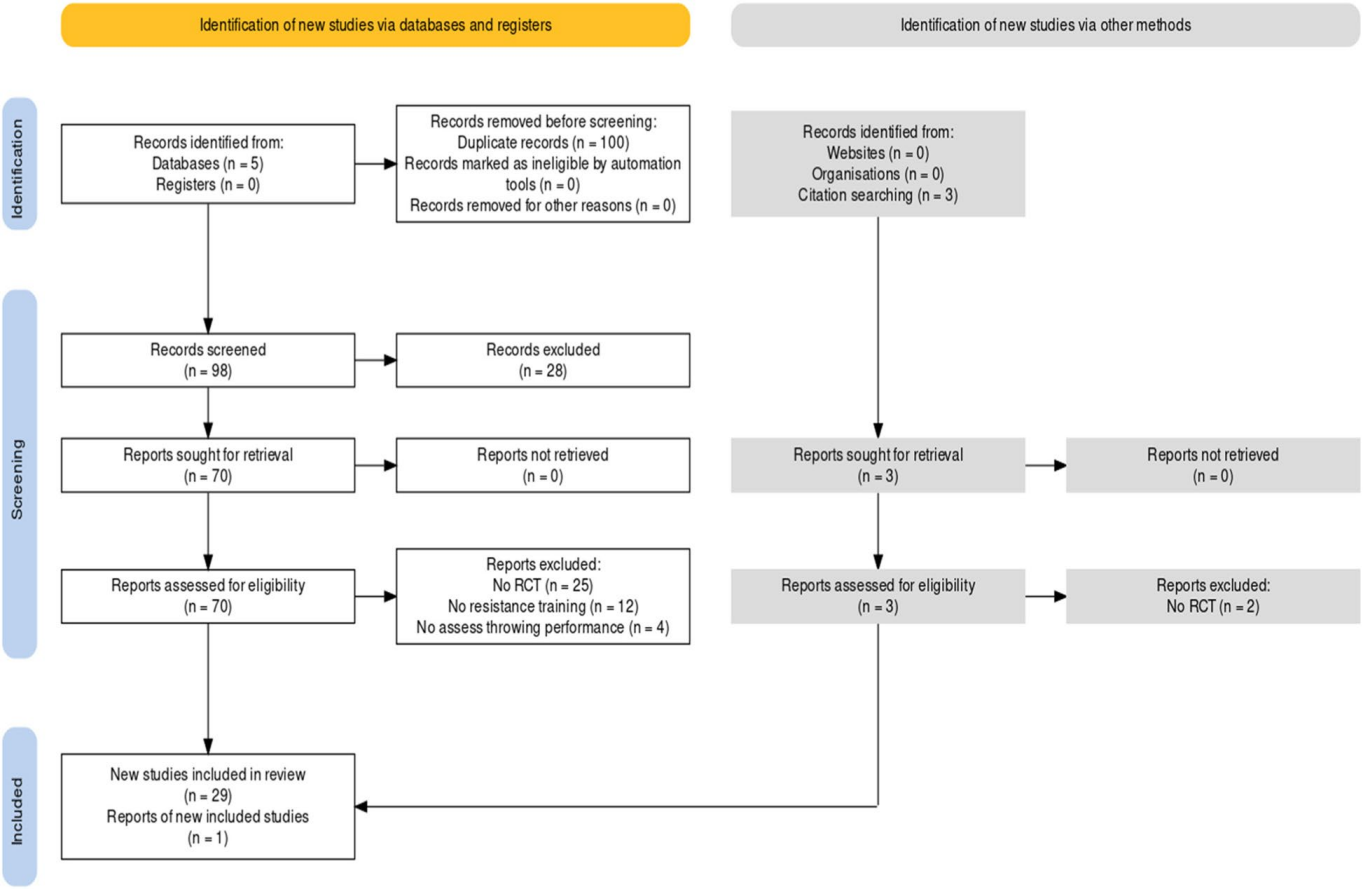
Flow chart of the included studies

Initially, there were identified 198 articles and following the removal of duplicates leaving 98 potential studies (Fig. 1). A total 28 articles were excluded because the title or abstract was not relevant with the inclusion criteria. Following these 70 full text articles were screened according to the inclusion criteria and 30 of them finally selected for final analysis.

### Methodological quality

#### Pedro score

Quality assessment scores for the included studies ranged between 6 and 8 (Table 1). Percentage scores ranged between 60 and 80% (median = 70%). Twenty-eight out of thirty studies were rated as high methodological quality studies (> 70%) while the rest of the studies were rated as moderate methodological quality studies (≤ 60%).

**Table 1:** Methodological quality scores of all studies

Reference	1	2	3	4	5	6	7	8	9	10	11	Total score
Hoff & Almasbakk (1995)	0	1	1	1	0	0	0	0	1	1	1	6/10
Sabido et al. (2016)	0	1	1	1	0	0	0	1	1	1	1	7/10
Loken et al. (2021)	0	1	1	1	0	0	0	1	1	1	1	7/10
Mancado et al. (2017)	0	1	1	1	0	0	0	1	1	1	1	7/10
Kuhn et al. (2018)	1	1	1	1	0	0	1	1	1	1	1	8/10
Maroto-Izquierdo et al. (2020)	0	1	1	1	0	0	0	1	1	1	1	7/10
Hermassi et al. (2010)	0	1	1	1	0	0	0	1	1	1	1	7/10
Abuajwa et al. (2022)	1	1	1	1	0	0	0	1	1	1	1	7/10
Madruga-Parera et al. (2022)	1	1	1	1	0	0	1	1	1	1	1	8/10
Hermassi et al. (2015)	0	1	1	1	0	0	0	1	1	1	1	7/10
Ignjatovic et al. (2012)	0	1	1	1	0	0	0	1	1	1	1	7/10
Raeder et al. (2015)	0	1	1	0	0	0	0	1	1	1	1	6/10
Hammami et al. (2020)	0	1	1	1	0	0	0	1	1	1	1	7/10
Van Den Tillar et al. (2020)	0	1	1	1	0	0	0	1	1	1	1	7/10
Ettema et al. (2008)	0	1	1	1	0	0	0	1	1	1	1	7/10
Hermassi et al. (2019c)	1	1	1	1	0	0	0	1	1	1	1	7/10
Hermassi et al. (2019d)	1	1	1	1	0	0	1	1	1	1	1	8/10
Ozmen et al. (2020)	1	1	1	1	0	0	0	1	1	1	1	7/10
Aloui et al. (2019)	1	1	1	1	0	0	0	1	1	1	1	7/10
Bauer et al. (2021)	1	1	1	1	0	0	0	1	1	1	1	7/10
Hammami et al. (2022)	0	1	1	1	0	0	0	1	1	1	1	7/10
Kusuwamati et al. (2022)	0	1	1	1	0	0	0	1	1	1	1	7/10
Mascarin et al. (2017a)	1	1	1	1	0	0	0	1	1	1	1	7/10
Mascarin et al. (2017b)	1	1	1	1	0	0	0	1	1	1	1	7/10
Genevois et al. (2014)	1	1	1	1	0	0	1	1	1	1	1	8/10
Hermassi et al. (2011)	0	1	1	1	0	0	0	1	1	1	1	7/10
Hermassi et al. (2019a)	0	1	1	1	0	0	1	1	1	1	1	8/10
Hermassi et al. (2019b)	0	1	1	1	0	0	1	1	1	1	1	8/10
Liu & Li (2021)	0	1	1	1	0	0	0	1	1	1	1	8/10
Bouagina et al. (2022)	1	1	1	1	0	0	0	1	1	1	1	7/10

1 Criterion satisfied, 0 Criterion not satisfied

### Study characteristics

The characteristics of the included studies are presented in Tables 2 and 3. A total of 727 handball players (males = 388, females = 292) were participated. The sample size of participants in these studies ranged from eleven [10] to forty-two subjects [21]. Τhe average number and the age of the participants of all studies was 25.1 (± 7.18) and 14.9 to 23.4 years old respectively. Ten studies included adolescent athletes [11, 17, 19–21, 23, 25, 31, 55, 56]. Time spent playing handball ranged from 2.7 to 16.0 years while ten studies did not report this information [13, 15, 17, 22, 25, 26, 28, 31, 57, 58].

**Table 2:** Types and protocols of resistance exercises that were used in the included studies

Study	RT modalities	Duration	Frequency	Sets and Reps/duration	Intensity (% of 1RM or other)	Between set rest	Notes
Hoff & Almasbakk (1995)	Barbell training	9 weeks	2/week	3 × 5 or 6	85% of 1 RM	2–5 min	Bench press with maximum speed Concentric
Sabido et al. (2016)	Barbell training	4 weeks	2/week	4 × 6	2 × 30%, 2 × 50%, 2 × 70% of 1RM in each set	3 min	Bench press. Plyometric movement with known load (one group) or unknown load (other group)
Loken et al. (2021)	Barbell training	8 weeks	2/week	3 × 5 (1st week), 3 × 4 (2nd week), 3 × 3 (3rd week),	40% (1st week), 50% (2nd week), 60% (3rd week) of 1 RM	3 min	Bench press with lightly touch the chest (one group) or with ballistic movement (other group)
Abuajwa et al. (2022)	Barbell training	5 weeks	3/week	4 × 6	60% (1st group), 40 (2nd group) of 1 RM	Not reported	Bench press with low speed (one group) or high speed (other group)
Manchado et al. (2017)	Core training	10 weeks	3/week	Not reported	154–170 ECOs (1–3 weeks), 176–228 ECOs (4–7 weeks), 228–238 ECOs (8–10 weeks)	Not reported	7 exercises each session (rectus abdominis, external and internal oblique muscles, lumbar and gluteal muscles). Weeks 1–3 (low difficulty exercise, 20 min), weeks 4–7 (medium difficulty exercises, 15–20 min, weeks 8–10 (medium to high level exercises, 20–25 min)
Kuhn et al. (2018)	Core training	6 weeks	2/week	2 × 45 s each exercise	3—8 kg	1 min	Core and rotational exercises. Progression: Static to dynamic movements, low load to high load, low level of instability to high level of instability
Ozmen et al. (2020)	Core training	6 weeks	2/week	20 s (isometric exercises) and 20 reps (isotonic exercises)	Progressively increase difficulty of exercise	Not reported	Exercises focusing in abdominal, pelvic and low back muscles
Maroto-Izqierdo et al. (2020)	Weight machine training	6 weeks	2/week	4 × 7	Not reported	Not reported	Flywheel or Pneumatic device. Lateral raise, internal and external shoulder rotation with a flywheel device (one group) or with a pneumatic device (other group)
Hermassi et al. (2010)	Barbell trainingRT	10 weeks	2/week	2 to 4 × 3 to 6 (moderate resistance training), 3 to 6 × 1 to 3 (heavy resistance training)	55–75% of 1RM (moderate resistance), 80–95% of 1RM (heavy resistance)	1–1.5 min (moderate resistance), 3–4 min (heavy resistance)	2 exercises (pull over and bench press). Subjects in heavy resistance training group performed the exercises at a slow velocity, while the subjects in moderate resistance training group performed the exercises as rapidly as possible
Madruga-Parera et al. (2022)	Weight machine training)	8 weeks	2/week	3 × 8 to 12	6 to 9 of scale rated of perceived effort	Not reported	Acceleration, Lateral squat (stage 1), Single leg hop, Acceleration, Lateral lunge (stage 2), Crossover step, Acceleration, Lateral step & unilateral countermovement jump (stage 3), crossover step, acceleration, 180° turn (stage 4)
Hermassi et al. (2015)	Medicine ball training	8 weeks	3/week	2 to 4 × 75 to 85 throws (regular throwing), 2 to 4 × 10 to 20 throws (medicine ball throwing)	3 kg medicine ball	2 min	Overhead throws with the medicine ball (one group) or with normal ball (other group)
Ignjatovic et al. (2012)	Medicine ball training	12 weeks	2/week	3 × 10 to 30 throws	1 to 3 kg medicine ball	10 to 30 s	Medicine ball exercises in standing, sitting and lying position and with jump
Raeder et al. (2015)	Medicine ball training	6 weeks	3/week	3 × 6 to 10 throws (medicine ball throwing), 1 × 8 to 12 throws (regular throwing)	1 to 2 kg medicine ball	60 to 90 s (medicine ball throwing), 5 s (regular throwing)	Exercises focusing in overhead throw, side throw, combination of throw with squat and single-arm throw
Hammami et al. (2020)	Plyometrics	10 weeks	2/week	10 × 6 (upper limb), 2 to 3 × 6 (lower limb), 10 s sequence each exercise	60 to 90 contacts number	30 s	Push up, hurdle jump, lateral hurdle jump, stretched leg jump, jump with 180 rotation, horizontal jump
van Den Tillar et al. (2020)	Plyometrics	12 weeks	2/week	2 to 5 × 8 to 30 jumps (plyometric training), 3 × 6 squats (strength training)	156–195 jumps per session (plyometric training), 40–45% of 1RM (strength training)	Not reported	Plyometric-strength to strength-plyometric after 6 week of training
Ettema et al. (2008)	Weight machine training	8 weeks	3/week	3 × 6 (pulley device throwing), 81 throws with normal ball (regular throwing)	85% of 1RM with 27.8 Newtons (pulley device throwing)	Not reported	Pulley device mimicking throwing. Both groups performed the experimental training sessions in combination with normal handball training
Hermassi et al. (2019a)	Barbell training	8 weeks	2/week	3 to 4 × 3 to 8	55 to 85% of 1RM (weekly increase)	3 min	4 exercises (clean and jerk, bench press, snatch, pull-over)
Hermassi et al. (2019b)	Barbell training	10 weeks	2/week	3 × 5 to 10	60% of 1RM (first 2 weeks), 70% of 1RM (week 3 to 5), 75–85% of 1RM (week 5 to 10)	3 min	4 exercises (clean and jerk, bench press, snatch, half-squat) with 2 weeks tapering intensity by 60%
Hermassi et al. (2019c)	Barbell training	10 weeks	2/week	3 to 5 × 6 to 10 (squat, bench press, pull over), 2 × 10 to 30 throws (medicine ball), 4 to 5 × 5 to 10 (drop jumps), 4 × 16 to 20 (hurdle jumps) 1 to 4 × 8 to 14 (one legged balance training)	50—75% of 1RM (squat, bench press, pull over), 45 to 60 cm (drop jumps), 50 to 70 cm (hurdle jumps), 3 kg medicine ball	Not reported	Bench press, squat, pull-over with or without Handball specific drills. Handball drills consists of ball shots and crossing exercises and lasted 30–45 min
Hermassi et al. (2019d)	Circuit training	10 weeks	2/week	2 × 6 to 12 (frontal jumps over barrier), 3 × 6 to 8 (bench press, pull-over), 3 × 4 to 10 (squats), 2 × 8 to 12 (frontal sprint), 4 × 16 to 20 (hurdle jumps), 4 to 12 × 5 (zig zag sprint), 4 × 5 to 10 (depth jumps)	40–60 cm height (frontal jumps over barrier), 60–80% of 1RM (bennch press, squats, pull-over), 10–20 m (frontal sprint), 30–50 cm height (depth jumps)	3 min	Progression with decreasing the rest intervals between sets. The duration of the circuit training was 30–35 min
Aloui et al. (2019)	Elastic band training	8 weeks	2/week	3 × 12 to 15	black elastic band 250% elongation-78.4 N (1–4 sessions), silver elastic band 250% elongation-113 N (5–8 sessions), gold elastic band 200% elongation-149 N (9–12 sessions), gold elastic band 250% elongation-178 N (13–16 sessions)	90 s	3 exercises for shoulder focusing in internal rotation and adduction and 1 exercise for elbow focusing in extension
Bauer et al. (2021)	Elastic band	9 weeks	3/week	3 × 8 (weeks 1, 4 and 7), 3 × 10 (weeks 2, 5 and 8), 3 × 12 (weeks 3, 6 and 9)	Green elastic band 4.6 libres and 100% elongation	Not reported	All elastic band exercises were performed to the shoulder joint
Hammami et al. (2022)	Elastic band training	10 weeks	2/week	3 to 5 × 10	Red elastic band (week 1), green elastic band (week 2–4), blue elastic band (week 5–7), black elastic band (week 8–10). 250% elongation in all bands	30 s	8 exercises (flies, row with high elbows, trunk rotation, standing press, knee extension, knee flexion, half-squat, hip adduction),
Kusuwamati et al. (2022)	Elastic band training	8 weeks	3/week	Not reported	Light resistance	Not reported	Exercises for the forearms
Mascarin et al. (2017a)	Elastic band training	6 weeks	3/week	3 × 10 to 20	blue elastic band (lower resistance), black, silver and gold elastic band (greater resistance,	30 s	Shoulder internal rotation from 90˚ shoulder abduction and in neutral position. Progressively increase difficulty of the exercise with the number of set repetitions, the band colour and borg scale
Mascarin et al. (2017b)	Elastic band training	6 weeks	3/week	3 × 10 to 20	blue elastic band (lower resistance), black, silver and gold elastic band (greater resistance,	30 s	Shoulder external rotation from 90˚ of shoulder abduction and in neutral position. Progressively increase difficulty of the exercise with the number of set repetitions, the band colour and borg scale
Genevois et al. (2014)	Body weight training	6 weeks		4 × 6 repetitions (week 1–5) and 5 × 6 (week 6)	115 to 95 cm distance from wall (scapular retraction with external rotation), 105 to 85 cm distance from wall (scapular retraction)	90 s	TRX exercises (one group) or regular training (other group) After 6 weeks the program was reserved between groups
Hermassi et al. (2011)	Barbell training	8 weeks	2/week	3 × 3 to 5	90% of 1 RM (week 4–5), 95% of 1RM (week 6–8),	3–4 min	3 exercises (bench press, pull-over, half-back squat) with low velocity contractions
Liu & Li (2021)	Functional training	8 weeks	3/week	4 × 10 to 15 (isotonic exercises), 4 × 60 s (isometric exercises)	20—40% ratio	60—120 s	Functional recovery and movement mode correction (2 weeks), functional improvement and special advancement stage (4 weeks), functional stability and special promotion stage (2 weeks)
Bouagina et al. (2022)	Weight machine training	8 weeks	2/week	8 repeated consecutive sequences of ball propulsion	Not reported	3 min	Arm/shoulder specific strength device. Progression made by increasing the number of sets the first 3 weeks. In order to decrease the muscle damage, the sets was decreased the 4th week

**Table 3:** Characteristics of the included studies

*Authors*	Study type	Sample	Groups	Outcome measures	Results
*Hoff & Almasbakk (1995)*	RCT	11 females competitive handball players from second division	1)Barbell training (*n* = 6) 2) Traditional handball training (*n* = 5)	1)Standing throw velocity 2)Running throw velocity (Video-camera)	Barbell training group had a greater improvement only in running throw compared to control group
*Sabido *et al. *(2016)*	RCT	28 junior team handball players	1)Barbell training with unknown load 2)Barbell training with known load 3)Traditional handball training	1)Standing throw velocity 2)Jumping throw velocity (radar gun)	The barbell training group with unknown loads showed a greater improvement in both types of throws compared to the other two groups
*Loken *et al. *(2021)*	RCT	16 males amateurs handball players	1)Barbell training without bounce(*n* = 8) with traditional handball training 2)Barbell training with bounce (*n* = 8) with traditional handball training	1)Standing throw velocity 2)Running throw velocity (radar gun)	Both resistance training groups showed significant improvement in the standing throw velocity. The group without bounce showed greater improvement in running throw velocity compared to the group with bounce
*Manchado *et al. *(2017)*	RCT	30 males handball players	1)Core training (*n* = 15) 2) Traditional handball training (*n* = 15)	1)Standing throw velocity 2)Running throw velocity 3)Jumping throw velocity (radar gun)	Core training group showed a greater improvement in all types of throwing velocity than the control group
*Kuhn *et al. *(2018)*	RCT	20 non-elite females handball players	1)Core training(*n* = 10) 2 Traditional handball training (*n* = 10)	1)Standing throw velocity 2)Jumping throw velocity (opto-electric timing system)	No statistically significant differences were found in standing throw between the core training and control group. A significant improvement in jumping throw was observed in both the core training and control group
*Maroto-Izquierdo *et al. *(2020)*	RCT	18 males elite handball players	1)Weight machine training using iso-inertial flywheel training (*n* = 9) 2)Weight machine training using pneumatic resistance training (*n* = 9)	1)Standing throw velocity 2)Sitting throw velocity (radar gun)	A significant improvement in standing throw and sitting throw velocity was found in both iso-inertial flywheel training group and pneumatic resistance training group. There were no statistically significant differences between groups
*Hermassi *et al. *(2010)*	RCT	26 males elite handball players	1)Barbell training with heavyresistance (*n* = 9) 2)Barbell training with moderate resistance (*n* = 9) 3) Traditional handball training (*n* = 8)	1)Standing throw velocity 2)Running throw velocity (digital video-camera)	The heavy resistance training group showed a significant improvement in standing throw and running throw compared to control group. The moderate resistance training group showed a significant improvement only in running throw compared to control group
*Abuajwa *et al. *(2022)*	RCT	22 males active collegiate handball players	1)Barbell training with low-movement velocity 2)Barbell training with high-movement velocity	Standing throw velocity (wearable wireless accelerometer)	A significant increase in throwing velocity was observed in the low-movement velocity group and high-movement velocity group compared to baseline. There were no significant differences between groups
*Madruga-Parera *et al. *(2022)*	RCT	34 young males handball players	1)Weight machine training using Isoinertial resistance training (*n* = 17) 2)Weight machine training using cable resistance training (*n* = 17)	Standing throw velocity (radar gun)	Isoinertial resistance training group showed a greater improvement in throwing velocity than cable resistance training group
*Hermassi *et al. *(2015)*	RCT	34 elite males handball players	1)Medicine ball training (*n* = 12) 2)Regular throwing training (*n* = 12) 3) Traditional handball training (*n* = 10)	1)Standing throw velocity 2)Running throw velocity 3)Jumping throw velocity (digital video-camera)	The medicine ball training group significantly increased throwing velocity in all types of throws The regular throwing training group improved only in the jumping throw. There were no significant changes in throwing velocity in the control group
*Ignjatovic *et al. *(2012)*	RCT	21 young females handball players	1)Medicine ball training (*n* = 11) 2)Traditional handball training (*n* = 10)	Various medicine ball throws	The medicine ball training group showed a statistically significant increase in all medicine ball throwing tests than control group
*Raeder *et al. *(2015)*	RCT	28 competitive amateur females handball players	1)Medicine ball training (*n* = 15) 2)Traditional handball training (*n* = 13)	1)Standing throw velocity (radar gun) 2)Throwing accuracy (target mark)	The medicine ball training group showed significantly higher throwing velocity compared to the control group. No significant differences were found in throwing accuracy
*Hammami *et al. *(2020)*	RCT	34 young females elite handball players	1)Plyometric training (*n* = 17) 2)Traditional handball training (*n* = 17)	Medicine ball sitting shot put throw test	Both groups significantly increase medicine ball throw compared to baseline. Plyometric training improved more (27.6 vs 8.2% on average)
*Van Den Tillar *et al. *(2020)*	RCT	42 competitive males (n = 30) and females (12) adolescents handball players	1)Strength-Plyometrics (*n* = 21) 2)Plyometrics-Strength (*n* = 21)	1)Standing throw velocity 2)Running throw velocity (radar gun)	There was no significantly differences in throwing velocity between groups
*Ettema *et al. *(2008)*	RCT	13 females experienced handball players	1)Weight machine training using pulley device mimicking throwing (*n* = 7) 2)Traditional handball training (*n* = 6)	Standing throw velocity (3-dimensional digital video movement analysis system)	Both groups significantly increase throwing velocity compared to baseline without significant differences between groups
*Hermassi *et al. *(2019c)*	RCT	22 males elite handball players	1)Barbell training plus handball specific drills (*n* = 12) 2) Barbell training (*n* = 12)	1)Jumping and running throw velocity (digital video-camera) 2)Medicine ball standing overhead throw test	Both groups significantly increase jumping throw velocity and running throw velocity without differences between them. No significant improvement was found in medicine ball throw in any of the groups
*Hermassi *et al. *(2019d)*	RCT	22 males elite handball players	1)Resistance type circuit training (*n* = 12) 2)Traditional handball training (*n* = 10)	1)Running throw velocity 2)Jumping throw velocity (digital video-camera)	The circuit-training group showed significant improvement in all types of throws. Control group showed a decrease in throwing velocity for both measures with negative effect sizes
*Ozmen *et al. *(2020)*	RCT	20 males adolescents handball players	1)Core training 2)Traditional handball training	1)Standing throw velocity 2)Running throw velocity 3)Jumping throw velocity (sports radar)	No significant difference was found for throwing velocity between groups
*Aloui *et al. *(2019)*	RCT	30 national adolescents males handball players	1)Elastic band training (*n* = 15) 2)Traditional handball training (*n* = 15)	1)Standing throw velocity 2)Running throw velocity 3)Jumping throw velocity (digital video-camera)	The elastic band training group demonstrated greater improvement in all three types of throws compared to the control group
*Bauer *et al. *(2021)*	RCT	22 males adolescents handball players	1)Elastic band training (*n* = 16) 2)Traditional handball training (*n* = 16)	Standing throw velocity (radar gun)	Elastic band training group showed a significant improvement in throwing velocity compared to the control group
*Hammami *et al. *(2022)*	RCT	26 young females handball players	1)Elastic band training (*n* = 13) 2)Traditional handball training (*n* = 13)	Medicine ball sitting shot put throw test	Both groups showed a significant improvement in the medicine ball sitting shot put throw test, but the elastic band training group had a higher increase compared to the control group
*Kusuwamati *et al. *(2022)*	RCT	28 males university handball players	1)Elastic band training (*n* = 14) 2)Traditional handball training (*n* = 14)	1)Standing throw velocity (radar gun) 2)Throwing accuracy (goal success)	The elastic band training group showed significantly greater improvement in throwing velocity compared to the control group. No significant differences in throwing accuracy were found between the elastic band training and the control group
*Mascarin *et al. *(2017a)*	RCT	39 females handball players	1)Elastic band training (*n* = 21) 2)Traditional handball training (*n* = 18)	1)Standing throw velocity 2)Jumping throw velocity (radar gun)	The elastic band training group showed a greater improvement in standing and jumping throws No differences were observed in the control group
*Mascarin *et al. *(2017b)*	RCT	25 young females handball players	1)Elastic band training (*n* = 15) 2)Traditional handball training (*n* = 10)	Standing throw velocity (radar gun)	The elastic band training group showed a significant improvement in throwing velocity compared to the control group
*Genevois *et al. *(2014)*	RCT (crossover)	25 elite female high school handball players	1)Body weight training using Sling exercise in regular training (6 weeks) + regular training (6 weeks) (*n* = 12) 2)Regular training (6 weeks) + Body weight training using Sling exercise in regular training (6 weeks) (*n* = 13)	Standing throw velocity (radar gun)	There were no significant differences in throwing velocity between groups
*Hermassi *et al. *(2011)*	RCT	24 elite males handball players	1)Barbell training with heavy resistance (*n* = 12) 2)Traditional handball training (*n* = 12)	1)Standing throw velocity 2)Running throw velocity 3)Jumping throw velocity (digital video-camera)	The heavy resistance training group showed significantly greater improvements in all types of throw compared to the control group
*Hermassi *et al. *(2019a)*	RCT	20 males elite handball players	1)Barbell training (*n* = 10) 2)Traditional handball training (*n* = 10)	1)Standing throw velocity 2)Running throw velocity 3)Jumping throw velocity (digital video-camera) 4)Medicine ball throw test	Weight lifting training group showed significantly greater improvement in throwing velocity in all types of throw and significantly higher values during the medicine ball throw test compared to the control group
*Hermassi *et al. *(2019b)*	RCT	20 males elite handball players	1)Barbell training (*n* = 10) 2)Traditional handball training (*n* = 10)	1)Jumping throw velocity 2)Running throw velocity (digital video-camera)	The weight lifting training group showed greater improvements in both types of throws compared to the control group
*Liu & Li (2021)*	RCT	20 females elite handball players	1)Functional training (*n* = 10) 2)Traditional handball training (*n* = 10)	Standing throw velocity (radar gun)	The functional training group showed a significantly greater improvement in throwing velocity compared to the control group
*Bouagina *et al. *(2022)*	RCT	26 males adolescents handball players	1)Weight machine training using arm/shoulder strength device (*n* = 15) 2)Traditional handball training (*n* = 11)	1)Standing throw velocity 2)Running throw velocity 3)Jumping throw velocity (radar gun)	The weight machine training group showed significantly greater improvements in all types of throws compared to the control group

The modalities of RT that were used in the studies (Table 2) was barbell training, (bench press, squat, clean and jerk, snatch etc.) [10–14, 28, 59–61], training with weight machines [22, 23, 29, 37], body weight exercises [56], core training [15–17], medicine ball training [38, 55, 62], plyometrics [20, 21], circuit training [57], elastic resistance band training [18, 19, 25, 26, 31, 58], and functional training [63]. The total duration of the resistance exercise program in the studies ranged between 4 and 12 weeks (median = 8). Sabido et al. [11] used the shorter duration (4 weeks), while 2 studies [20, 55] used a longer duration (12 weeks). Eighty per cent of the studies used as a control group the traditional handball training program. Two studies [12, 13] used as a control / comparison group other parameters of the same exercise and other two studies [22, 23] used a different type of exercise. In one study [21], after six weeks of training the groups swapped their training programme from plyometric-strength to strength-plyometric. Hermassi et al. [59] used the resistance exercise with or without handball specific drills. Fourteen studies assessed the throwing velocity with radar gun [11, 12, 15, 17, 19, 21–23, 25, 26, 56, 58, 62, 63], nine studies with digital video-camera [9, 10, 14, 35, 42, 45, 47, 48, 50], one study with 3-dimensional digital video movement analysis system [37], one study with wearable wireless accelerometer [13] and one study with opto-electric timing system [16]. Only two studies assessed the throwing accuracy [58, 62] and four studies the distance at the medicine ball throw test [10, 17, 25, 37] as measures of throwing performance.

### Jumping throw velocity

**Fig. 2:**
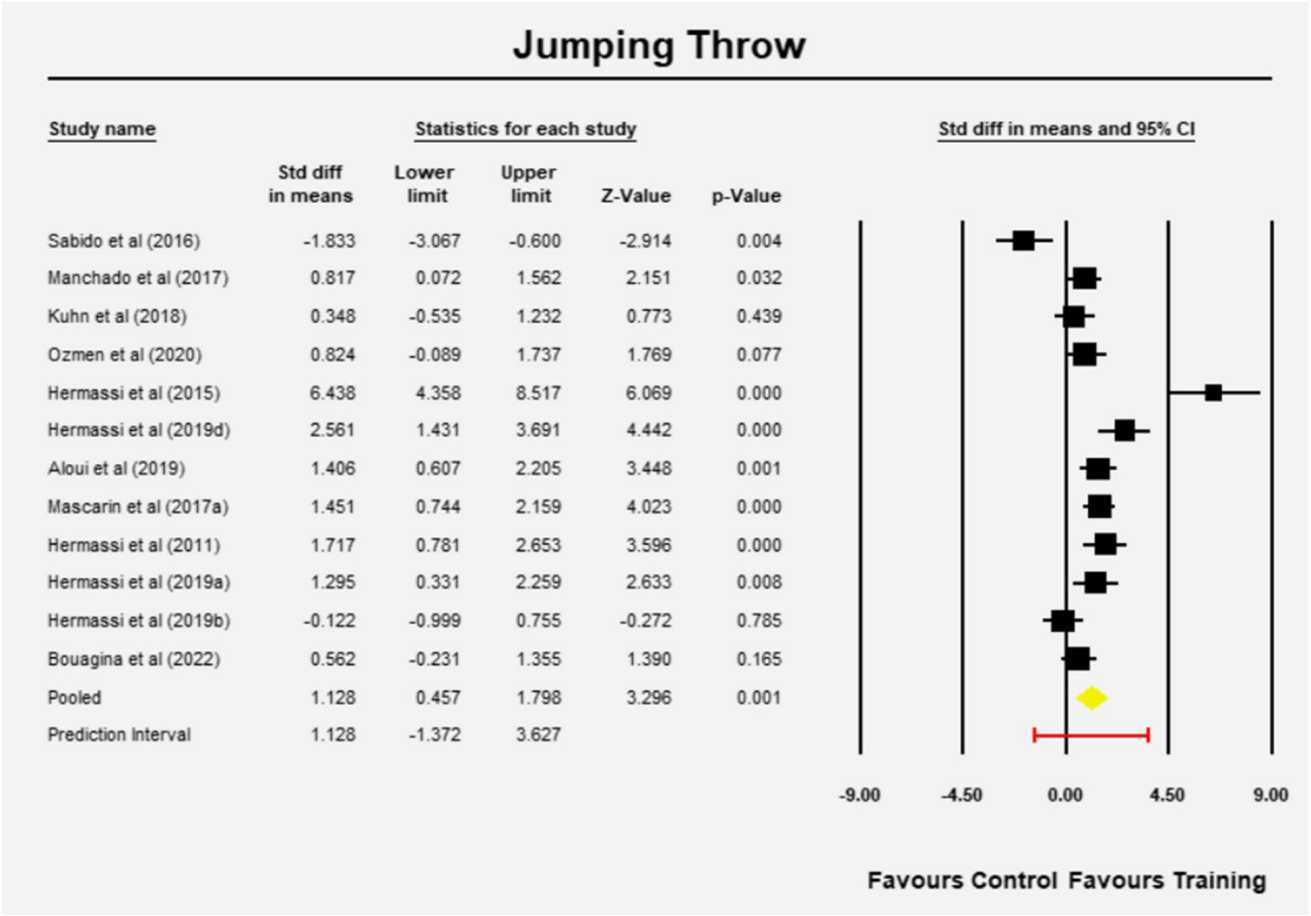
Mean effects of resistance training in Jumping throw velocity

The mean effect size is 1.128 with a 95% confidence interval of 0.457 to 1.798 (z = 3.296, *p* = 0.001) in favor of RT (Fig. 2). The Q-value is 68.961 with 11 degrees of freedom and *p* < 0.001. The I-squared statistic is 84%, indicating that 84% of the variance in observed effects reflects variance in true effects rather than sampling error. Tau-squared, the variance of true effect sizes, is 1.141 in d units. Tau, the standard deviation of true effect sizes, is 1.068 in d units. The prediction interval is -1.372 to 3.627 (Fig. 2).

**Fig. 3:**
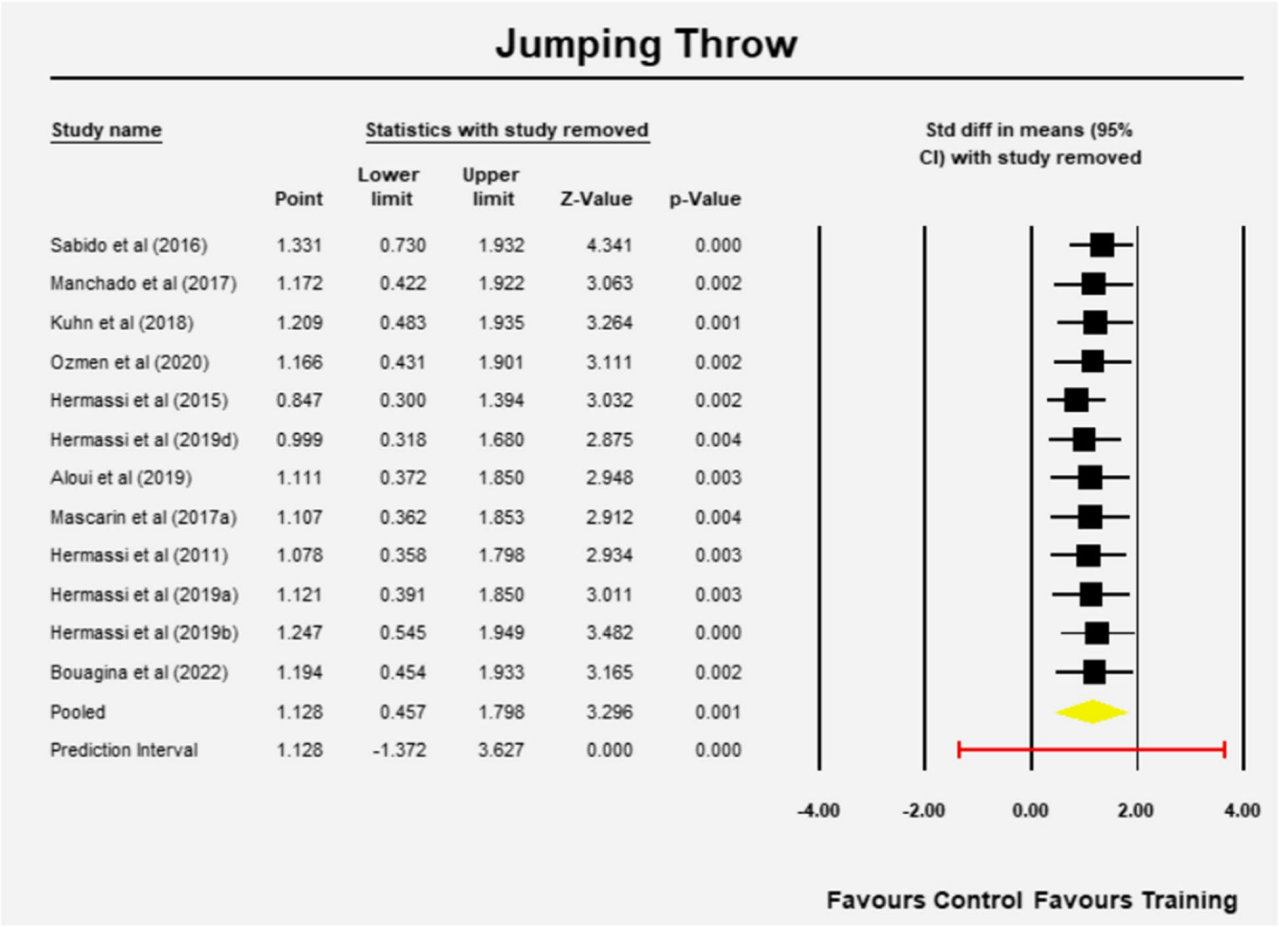
Sensitivity analysis Jumping throw (one study removed at a time)

Sensitivity analysis revealed that the results remained robust independent of the study removed (Fig. 3). Furthermore, a meta-regression on the methodological quality of the study showed no significant effect of the study quality on the outcome (Q = 0.09, df = 1, *p* = 0.765).

**Fig. 4:**
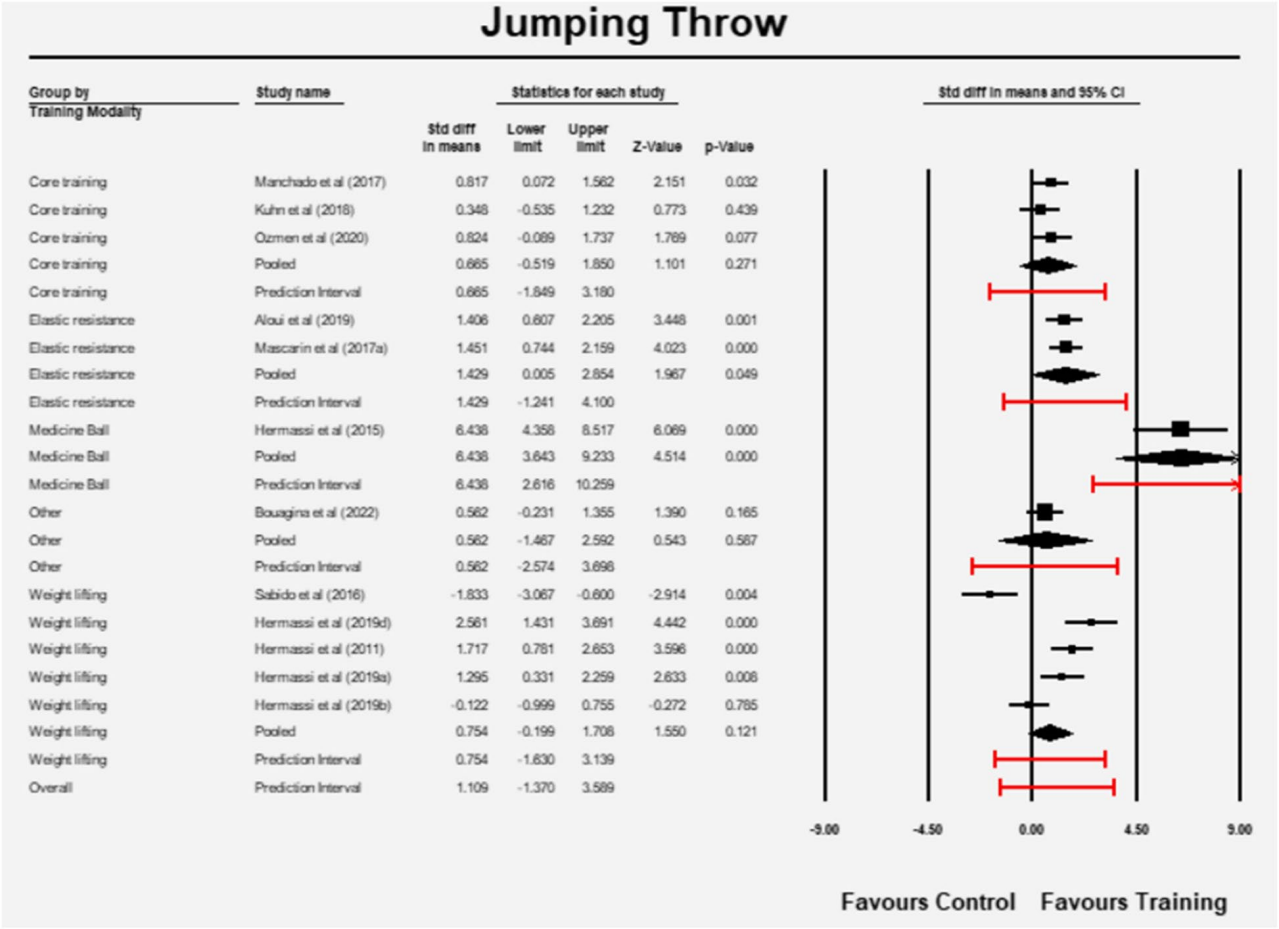
Effect sizes and prediction intervals between different subgroups (RT: resistance training)

Subgroup analysis revealed that the pooled estimates in the different subgroups were different (Q = 15.505, df = 4, *p* = 0.004). The mean effect size as well as the prediction interval for each subgroup is presented in Fig. 4. The 95% CI of the elastic resistance group and the medicine ball training group did not contain zero but results need to be interpreted with caution due to low number of studies in each of these groups (two and one respectively). Weight lifting (barbell training) did not seem to have an effect despite 6 studies were included in this subgroup. Similar observations were evident for the core training (3 studies) and the other subgroups (1 study).

### Running throw velocity

**Fig. 5:**
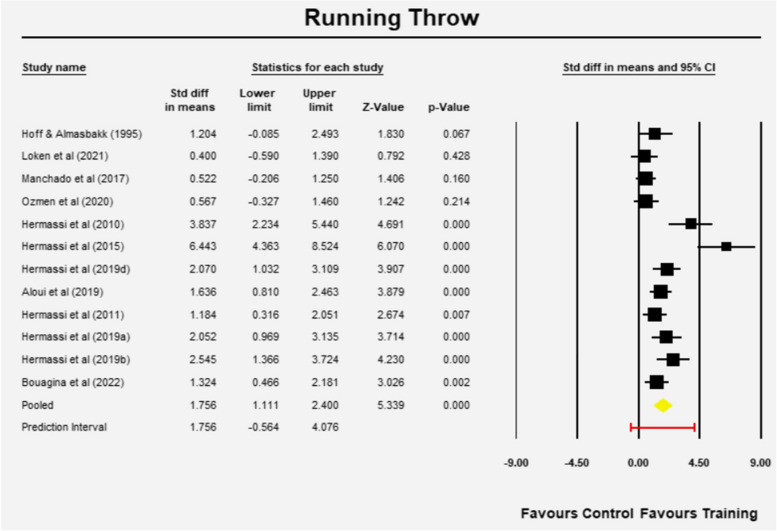
Mean effects of resistance training in Running throw velocity

The mean effect size is 1.756 (95%CI 1.111—2.400) (z = 5.339, *p* = 0.001) (Fig. 5). The Q-value is 51.57 with 11 degrees of freedom and *p* < 0.001. According to I-squared statistic, 79% of the observed variance can be attributed in variance of the real effects rather than sampling error. The variation of true effect sizes is 0.976 in d units as evident by Tau-squared. The standard deviation of true effect sizes is 0.988 in d units and the prediction interval is -0.564 to 4.076, which means the mean effect size might change with future studies (Fig. 5).

**Fig. 6:**
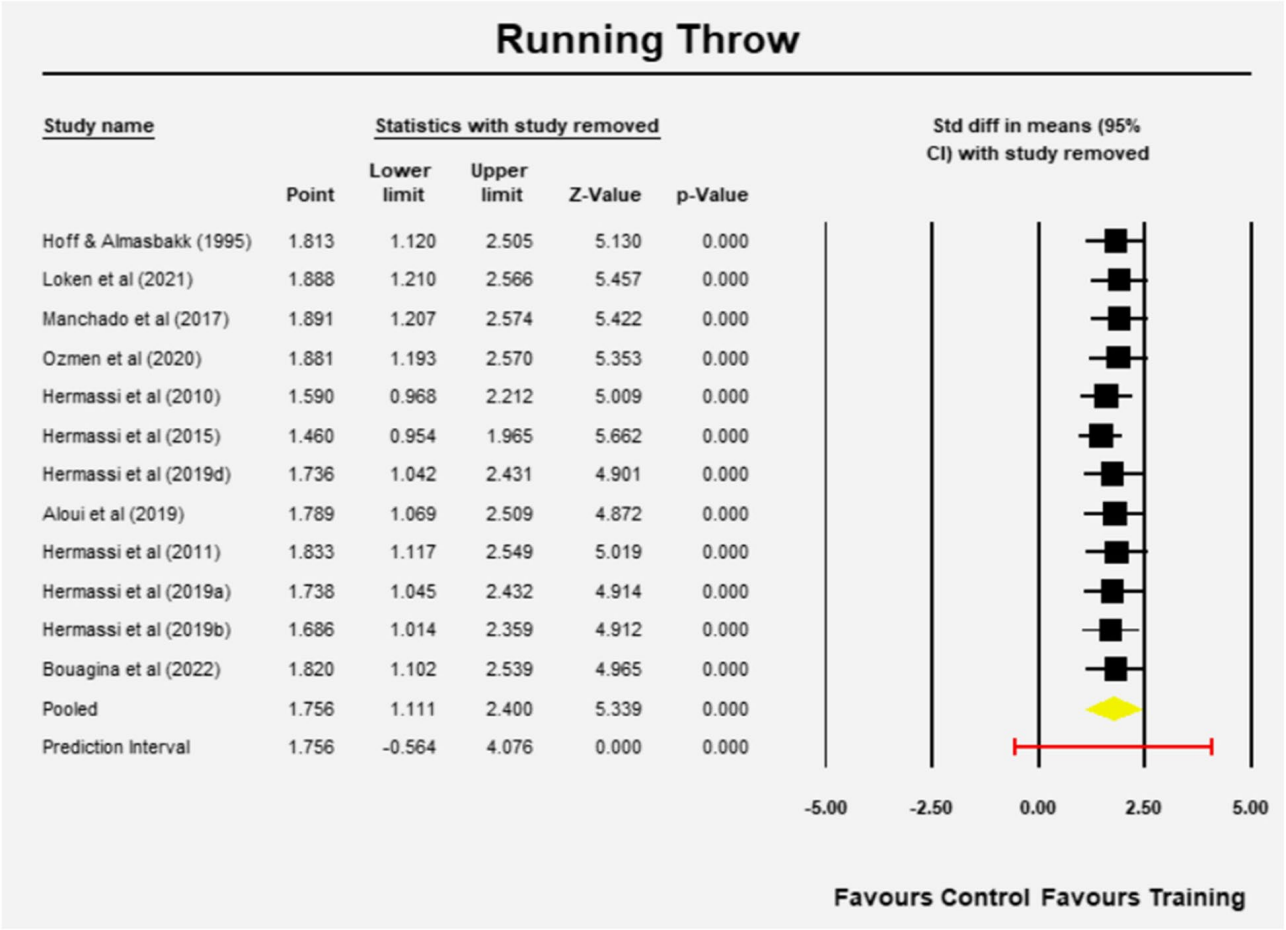
Sensitivity analysis Running throw (one study removed at a time)

Sensitivity analysis showed that the results remained the same regardless of which study was removed (Fig. 6). Furthermore, a meta-regression using methodological quality score as independent variable showed no significant effect of the study quality on the outcome (Q = 0.82, df = 1, *p* = 0.3652).

**Fig. 7:**
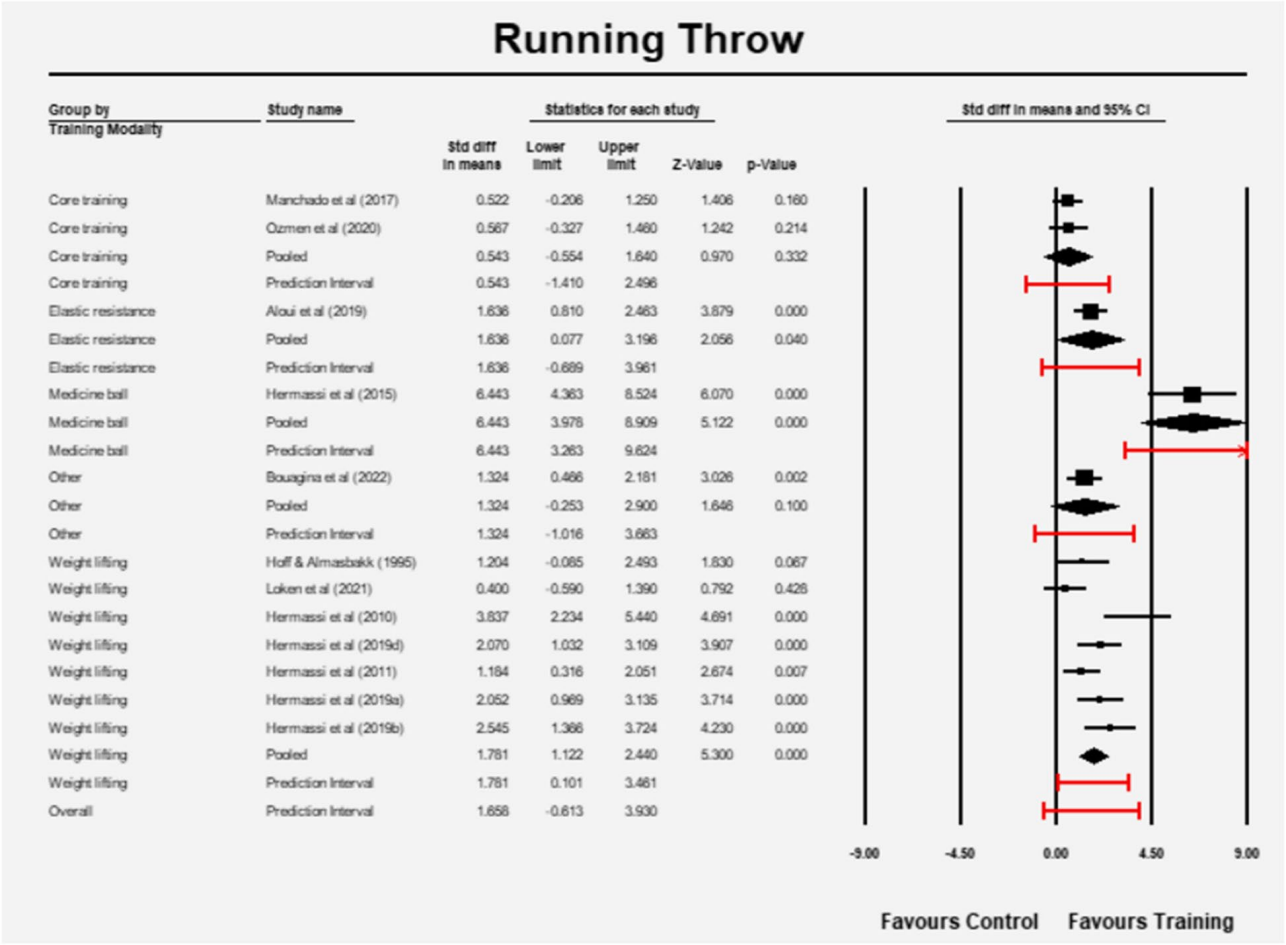
Effect sizes and prediction intervals between different subgroups (RT: resistance training)

The pooled estimates in the various subgroups differed, according to the subgroup analysis (Q = 18.750, df = 4, *p* = 0.001). Fig. 7 shows the mean effect size and the prediction interval for each subgroup. The elastic resistance, the medicine ball and the other subgroups contained only one study each, therefore the result is the same as the effect size of the study. Barbell training was utilized in 8 studies and seems to have a significant effect, as the 95% CI of both the estimated effect size and the prediction interval do not contain zero while core training contained only two studies and showed no significant effect.

### Standing throw velocity

**Fig. 8:**
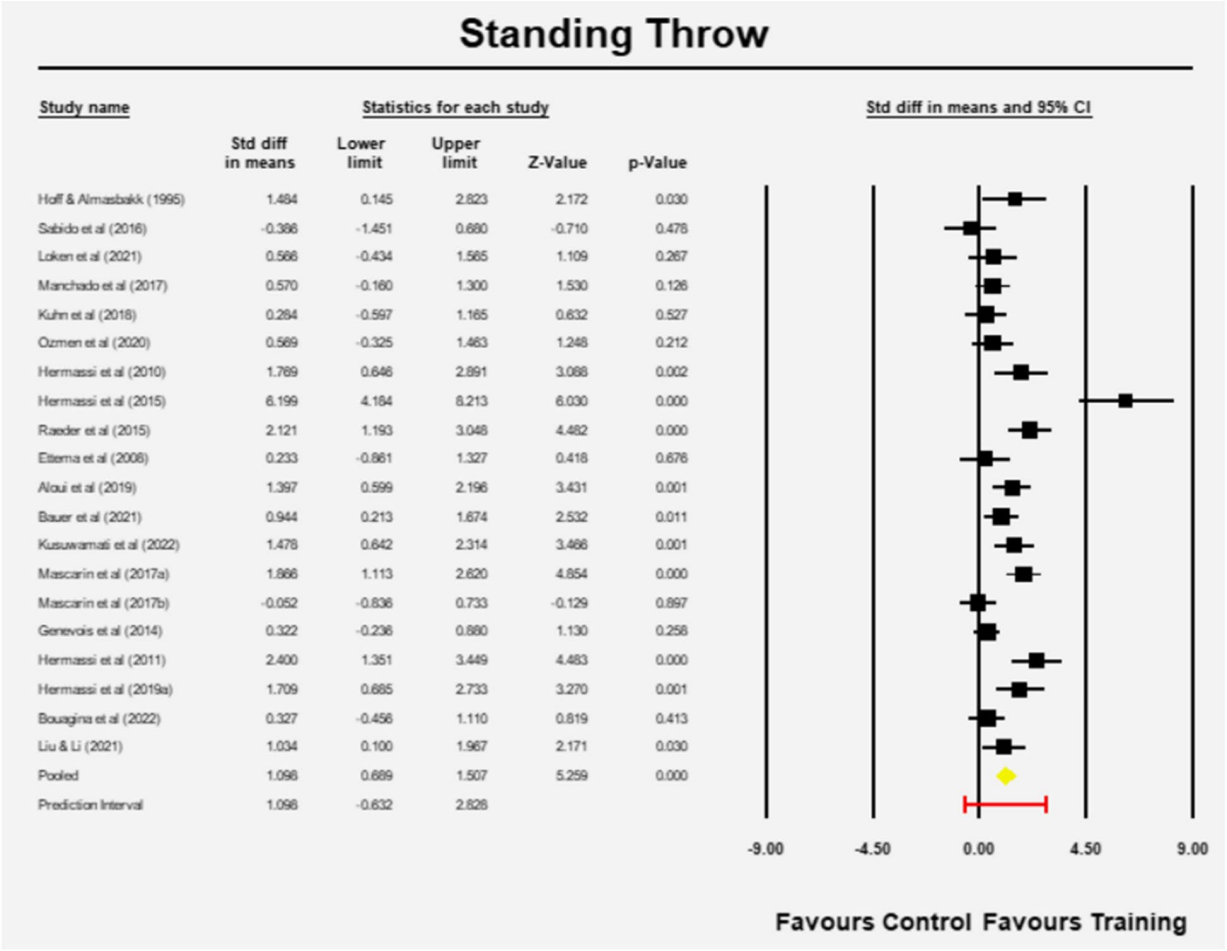
Mean effects of resistance training in Standing throw velocity

The estimated effect size is 1.098 (95% CI 0.689–1.507) with z = 5.259 and *p* = 0.001 in favor of RT (Fig. 8). The Q-value is 78.489 with 19 degrees of freedom and *p* < 0.001. The I-squared statistic indicates that 76% of the variance is due to variation in genuine effects and not sampling error. The variance and the standard deviation of true effect sizes are 0.634 and 0.796 in d units, respectively. The prediction interval is -0.632 to 2.828, which means the estimate has the potential to be misleading at times (Fig. 8).

**Fig. 9:**
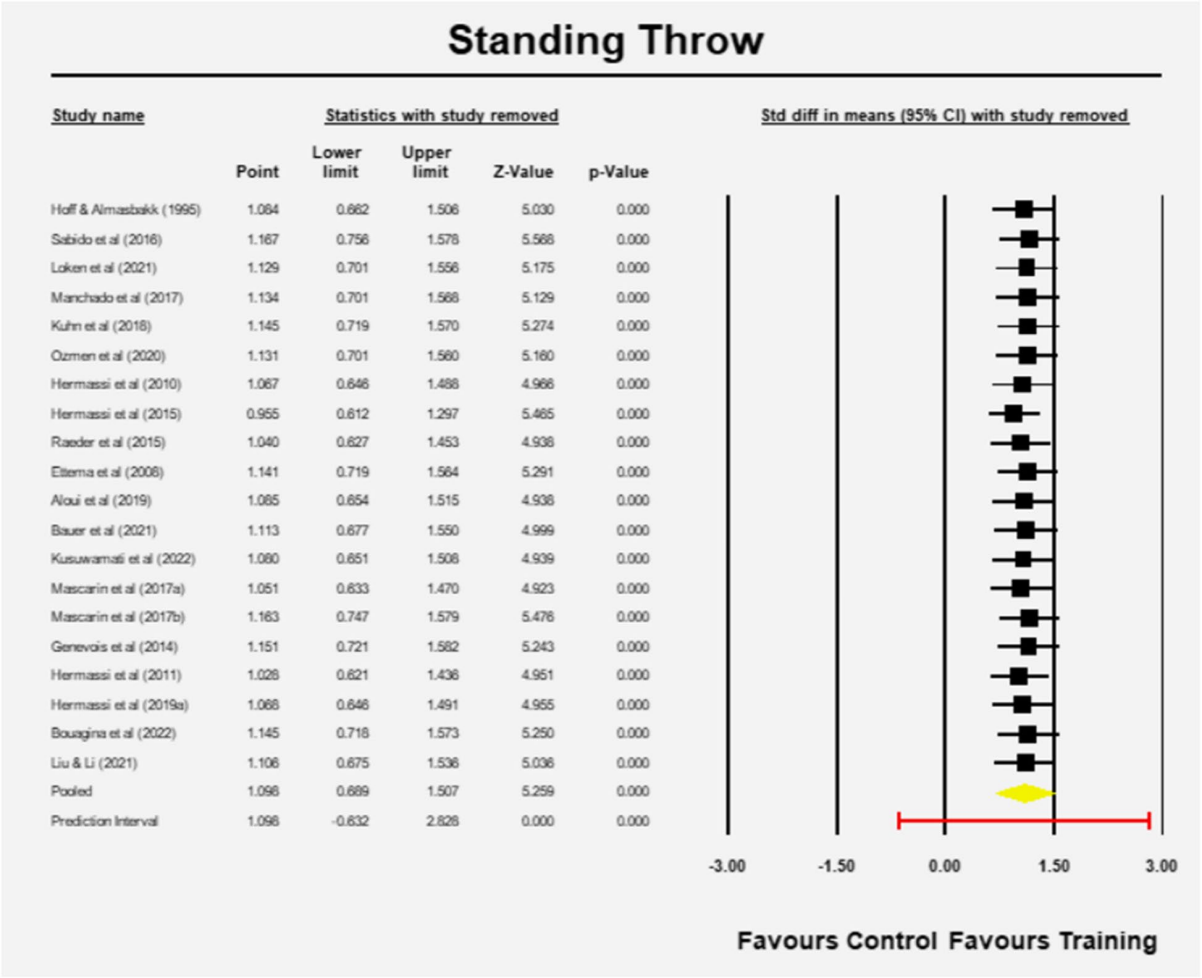
Sensitivity analysis Standing throw (one study removed at a time)

Sensitivity analysis revealed no effect of any particular study in the outcome (Fig. 9). Furthermore, the methodological quality score had no significant effect on the outcome based on the met-regression results (Q = 1.40, df = 1, *p* = 0.2362).

**Fig. 10:**
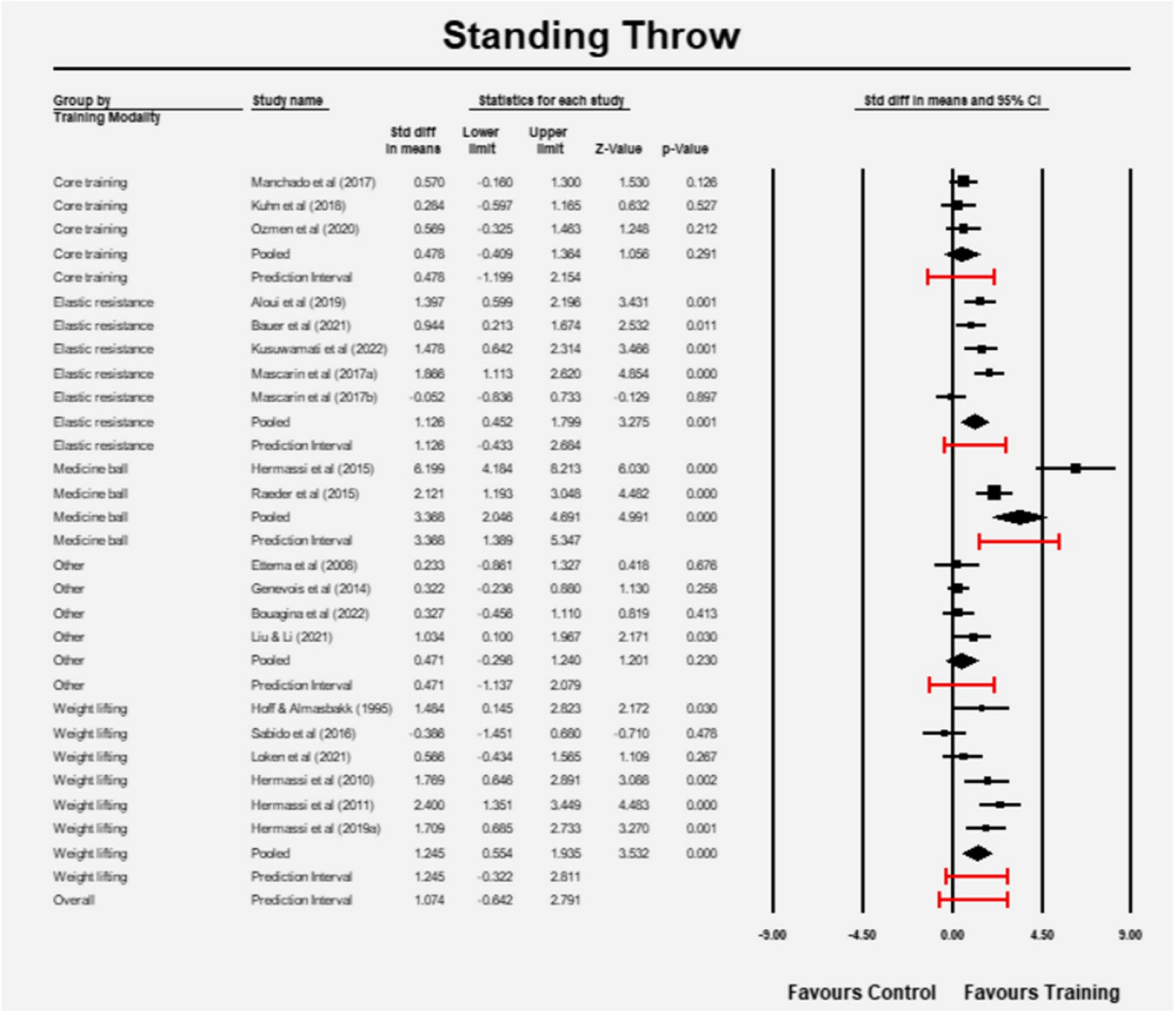
Effect sizes and prediction intervals between different subgroups (RT: resistance training)

Subgroup analysis differences between the various subgroups (Q = 15.918, df = 4, *p* = 0.003). The mean effect size and the prediction interval for each subgroup is illustrated in Fig. 10. The core training (3 studies) and the other (4 studies) subgroups had no significant effects. The barbell training (8 studies) and the elastic resistance subgroups (5 studies) seem to have a significant effect, but the prediction interval reveals the true effect is likely to be different. The medicine ball training seems to have a significant effect, but the pooled estimate and the prediction interval are based on two studies only and warrant attention.

### Throw distance

Five studies of high quality assessed the effect of resistance training in throw distance using one or more medicine ball throw tests. One study used medicine ball training, one used elastic resistance, one used plyometric training (upper and lower limb), one used weightlifting using barbell and the last one combined weightlifting (barbell) with handball specific drills. Limited evidence supports the use of all these practices to improve throw distance in handball.

### Throw accuracy and throw success

One study of moderate quality found no use of medicine ball training in improving throwing accuracy in handball compared to normal training (limited evidence). One study of high quality showed no benefit of elastic resistance training in improving throwing success in handball compared to standard training (limited evidence).

## Discussion

The aims of this systematic review and meta-analysis were: i) to investigate the level of evidence for the effect of resistance training on throwing performance in handball players; ii) to suggest recommendations for the appropriate resistance training program for the improvement of throwing performance.

Given its pivotal role in predicting the success or failure of overhead athletes, throwing velocity has emerged as a significant focal point in sports science research over the last decade [24, 64]. Resistance training has been shown to produce many benefits in different types of athletes, such as improvement of muscle strength, power, and muscle hypertrophy [65–67]. Since many overhead athletes generate their maximum throwing velocities through explosive rotational movements, a plethora of resistance training techniques [14, 18, 25, 37, 55, 56] have been investigated for their impact on velocity performance. The majority of the included studied used mainly elastic band training and barbell training. As a result, generalizing the data on RT modalities has been difficult..

### The effect of resistance training on throwing velocity

The key findings indicated that RT in general has a statistically significant effect on throwing velocity as shown by the mean estimate for all three throwing styles. The effect is higher in running throw and lower in standing throw. However, the prediction interval contained zero, in all three throwing styles and this means the outcome of future studies can show positive, negative, or no effect of resistance training on throwing velocity. The results suggest that based on current evidence RT is recommended as a method to increase throwing velocity but this recommendation might change with further research.

### The effect of resistance training modalities on jumping throw velocity

Furthermore, a strengthening program, of the shoulder internal rotator muscles in both adolescents and female handball players, using elastic resistance [18, 26], led to a significant increase in jumping throw velocity when compared to a control group. The studies used a duration of 6–8 weeks and a frequency of 2–3 times per week. The outcome is preliminary based on two high quality studies and the prediction interval suggests the true effect might be substantially different meaning future studies can show no effect, positive effect or even negative effect.

A possible explanation of this result is that maximal shoulder internal rotation during throwing is an important kinematic parameter to achieve a maximal ball velocity [6]. Previous studies found a significant correlation between the maximal angular velocity of internal rotation and ball velocity [6, 68]. It is worth noting that during the throwing motion, the shoulder internal rotator muscles have a frequent activation which has been linked to significant strength gains, leading to potential muscle imbalances between the internal and external rotator muscles [69]. Optimally, the strength ratio between external and internal rotator muscles should be 66% to 75% [69]. Moreover, the shoulder external rotator muscles are active as antagonistic muscles during the acceleration phase at the time of throwing. Due to this in the last phase of this action, they play a decisive role, which can affect the final output [70]. In addition, eccentric external rotation torques should be greater than concentric internal torques to overcome and decelerate not only the strength of the concentrically active internal rotators but also the other segmental forces associated with the dynamic nature of the throwing motion [71]. Therefore, it is crucial to include strengthening exercises for the entire rotator cuff muscle group, whether utilizing elastic bands or other exercise modalities. Additionally, it is important to include eccentric strengthening of the of the external rotator muscles as part of the regimen.

Furthermore, the medicine ball training group with overhead throws against a wall in addition to regular handball throwing was more effective for improving jumping throw velocity compared to regular handball throwing alone in elite males handball players [38]. This outcome is based on one high quality study therefore requires further confirmation. One possible explanation is that medicine ball training closely mimics the range of motion [72] and velocities commonly experienced in sports [73]. Nevertheless, these findings should be interpreted with caution because there is limited evidence available, and the number of studies that have investigated both modalities (elastic resistance and medicine ball training) is small. Additionally, barbell training [11, 28, 57, 60, 61] did not appear to have any effect, as the 95% CI of the pooled effect size contains zero. Similarly, for core training in three studies [15–17] and the additional subgroup (weight machine training) of one study [63], there was no effect on jumping throw velocity. Barbell training and core training might lack specificity to increase jumping throw velocity, although both address important elements of the kinetic chain.

Based on the prediction intervals of this outcome, none of the training modes examined can be recommended as «the optimal» way to increase jumping throw velocity. Future studies, perhaps, need to ‘tease’ load variables in more detail in order to optimize the outcome and help future recommendations.

### The effect of resistance training modalities on running throw velocity

Barbell training group (bench press, pull-over, clean and jerk, snatch, squat) [10, 12, 14, 28, 59–61] for 8 to 10 weeks with progressive (weekly or biweekly) increase in intensity from 60 to 95% of 1RM, 3 to 6 sets of adequate repetitions and rest intervals depending on the intensity, had a significant effect for improving running throw velocity. The result is based on findings from high quality studies (7/8). The mean effect and the prediction interval suggest the true effect varies by moderate to large effect but is still (at least marginally) beneficial. Therefore, this mode of training can be recommended if the aim is to increase running throw velocity. The result was based on male elite [14, 28, 59–61] and amateur handball players [12] and females competitive players [10]. These findings are in agreement with previous results of a systematic review [34], which showed that weight training with moderate and high intensity (> 55% of 1RM) was the best strategy to improve throwing velocity. The previous analysis included only elite players while the results of this systematic review generalize the effects of weight training beyond elite male handball players. In the case of experienced players, it's generally recommended to employ higher intensities, typically exceeding 80% of their one-repetition maximum to activate high-threshold, fast-twitch motor units [74]. Previous studies have shown that weight training produces superior strength-power adaptations compared to traditional resistance training [75, 76], jump training [77, 78] and kettlebell training [79]. During weight-lifting training the individuals performs ballistically movements with moderate to heavy loads which led to improvements in both velocity and power. As a result, neuromuscular adaptations may occur (i.e., motor unit recruitment, rate coding, etc.), which may improve strength-power characteristics [80]. Additionally, elastic resistance training [18], medicine ball training [38], and weight machine training [29] have a beneficial effect on running throw velocity. However, each of these subgroups consisted of just one study, indicating limited evidence. Consequently, further research is warranted in the future to expand our understanding in this area and to make additional recommendations.

### The effect of resistance training modalities on standing throw velocity

Barbell training [10–12, 14, 60, 61] seem to have a significant effect in increasing standing throw velocity. Studies used a duration of 8–10 weeks with a frequency of 2 times per week and intensities which increased gradually from 30 – 95% of 1-RM. The result is based on eight high quality evidence and diverse population such as amateur [12] and elite male [14, 60, 61] and female [10] handball players. Although the mean effect is statistically significant, the prediction interval suggests the true effect is likely to be substantially variable and can be non-significant. Therefore, this mode of training can not be recommended with the current evidence as the outcome of future studies might be positive, null or even negative.

In addition, a progressive elastic resistance training programme [18, 19, 25, 26, 58] has a significant effect in improving standing throw velocity in a mixed sample of male [18, 19, 58] and female [25, 26] handball players. The result is based on five high quality studies and the prediction interval suggests the true effect can be substantially different and not significant. Therefore, the recommendation of this mode of training is not possible based on the current evidence. Core training [15–17] along with the other subgroups [29, 37, 62, 63] did not exhibit statistically significant effects. This observation is drawn from the analysis of three studies in the case of core training and four studies for the other subgroups. Moreover, medicine ball training [38, 62] for 6–8 weeks and 3 times per week with 3–4 sets of 6–20 repetitions each exercise (overhead throw, backward throw, diagonal throw, rotational throw, shot-put throw) seem to have a significant effect in elite males [38] and amateur females handball players [62]. Although the pooled estimate and the prediction interval suggest the true effect is likely to be beneficial the calculations are based on two studies only. Therefore, caution is recommended with this finding.

### The effect of resistance training in throw distance

Five studies [20, 31, 55, 61, 62] of high quality assessed the effect of resistance training in throw distance using one or more medicine ball throw tests. One study used medicine ball training, one used elastic resistance, one used plyometric training (upper and lower limb), one used traditional weight training and the last one combined weightlifting with handball specific drills. Limited evidence supports the use of all these practices as a way to improve throw distance in handball due to limited number of the studies.

### The effect of resistance training in throwing accuracy

One study [62] of moderate quality found no benefit for medicine ball training in improving throwing accuracy in handball compared to normal training (limited evidence). One study [58] of high quality showed no benefit of elastic resistance training in improving throwing success in handball compared to standard training (limited evidence).

### Limitations

The current study had some limitations. First, only studies written in English language were included. Second, the heterogenetity of the studies also provided difficulty of the interpretation of the results and the derivation of solid suggestions. In addition, there's a chance that certain relevant papers were not included in our analysis, as our literature search was confined to publications up until 1995.

Furthermore, the studies included in this review suffer from several limitations. The transition of long-term muscular and physiological exercise adaptations are complicated due to short duration of resistance exercise program in some studies [11, 13, 16, 17, 22, 25, 26, 56, 62]. Previous research indicated that it takes at least 6 weeks of resistance training to increase motor unit synchronization [81]. Also, the parameters of the resistance exercise program (set, repetitions, between set rest, training modality) differed between the included studies. Also, the number of studies per training modality was low. These variables make reaching robust conclusions for recommendations difficult for the ideal resistance exercise program to improve throwing velocity. Some studies did not report sufficient details of the parameters of the program. For example, some studies used short rest time between sets [16, 18, 20, 25, 26, 38, 62], while other studies did not report the rest interval [13, 15, 19, 21–23, 37, 58, 59]. Research suggests that 2.5 to 5-min rest intervals resulted in a greater volume of work during a workout, greater ability to train with heavier loads and higher increase in strength compared to 0.5 to 2-min rest intervals [82, 83].

Moreover, none of the included studies used feedback tools aiming to increase the velocity. For example, velocity-based training was used in some studies as feedback and resulted in increases in velocity and power outputs up to 10% (77–79). Perhaps future studies can replicate this effect on handball players training to increase their throwing performance.

Ιn addition the included studies show heterogeneity in the methods of measuring throwing velocity (e.g. radar gun, digital video-camera, optoelectric timing system, wearable wireless accelerometer). This makes it more difficult to compare the results among studies.

Furthermore, other possible confounding factors (e.g.) that could have influenced results were not adequately controlled for. For example, some studies examined the influence of resistance training on throwing velocity in combination with traditional handball training. There is a possibility that the positive results did not come entirely from the resistance training. It is therefore necessary to use the results with a certain degree of caution. Αdditionally, some studies did not compare the effect of RT with traditional handball training alone, but rather with other modalities of RT. Due to this, it is challenging to definitively determine if combining resistance training with traditional handball training is more successful than traditional handball training alone.

The small sample size in some studies and in total can affect the generalization of the results. Also, there was no study comparing males to females or professional to amateur handball players in terms of the effect of resistance exercise in throwing performance. It remains unclear if there are significant differences in the effect of resistance exercise on throwing performance in these subgroups.

A proper throwing technique play an important role to achieve a higher ball velocity [84]. An incorrect throwing technique may be an important factor for the results. The throwing kinematic parameters that can affect the throwing ball speed are the proximal-to distal sequencing during the throwing motion [68], the optimal trunk and pelvis movement [6, 68], the maximal arm rotation [6], the maximal total elbow displacement [6], the velocity of body center of mass in the direction of the goal [6] and the efficient transfer of power from the lower body to the upper body, culminating in the release of the ball [85]. The strongest muscles of the lower limbs are responsible for the largest share of the overall impulse. Moreover, throwing velocity seems to be affected from the throwing technique. The highest throwing velocities are found in the running throw, followed by the standing throw, then the jumping throw and finally the pivot throw [68]. Hence, it is advisable to refine proper throwing technique as a precursor to strength training to enhance performance.

Most studies did not assess other important parameters of throwing performance, such as the throwing accuracy. Throwing accuracy is an important variable often associated with sporting success [86].

### Directions for future research

More studies are necessary that specifically investigate the effect of resistance training on throwing performance in handball players with longer duration of resistance exercise programs (> 6 weeks) and larger sample sizes. Furthermore, future studies should compare the effects of resistance training between specific handball subgroups (e.g. males vs females, younger vs older, elite players vs amateur players). The evaluation of the effect of resistance training in throwing accuracy needs to be incorporated and measured in future studies.

## Conclusions

Several RT techniques which focus to improve throwing performance in handball players were identified. Strong evidence was found only for the use of weight lifting training in increasing throwing velocity. Findings from the other resistance training modalities, including elastic resistance, medicine ball training, weight machine training, and core training, while yielding positive results, are limited impacting on the ability to reach firm recommendations. Furthermore, there is insufficient evidence to support the use of various training methods in increasing throw distance. Additionally, medicine ball training and elastic resistance training did not demonstrate any benefits in improving throwing accuracy.

**Table:** 

Practical Applications
• Weightlifting training in addition to traditional handball training for 8 to 10 weeks period and 2 times per week with progressive (weekly or biweekly) increase in intensity from 60 to 95% of 1RM is recommended to increase throwing velocity especially in running throw
• Higher intensities (> 80% of 1RM) are recommended in experienced players
• Medicine ball training with specific throwing exercises (overhead throw, backward throw, diagonal throw, rotational throw, shot-put throw) in addition to regular handball throw for 6–8 weeks and 3 times per week is a promising modality to increase velocity in all three throws but more evidence are needed before a solid recommendation can be advised
• Limited evidence supports the use of resistance training techniques as a way to improve throw distance or throwing accuracy in handball due to limited number of the studies

### Supplementary Information

**Supplementary Material 1.**

## Data Availability

The datasets generated and/or analysed during the current study are not publicly available but are available from the corresponding author on reasonable request (hadjisavvas.s@unic.ac.cy).

## Abbreviations

PRISMA: Preferred Reporting Items for Systematic reviews and Meta-Analyses
PICO: Patient/population, intervention, comparison, and outcomes
RT: Resistance Training
RM: Repetition Maximum
ECOs: Exercise Causality Orientation’s scale
BES: Best Evidence Synthesis
MeSH: Medical Subjects Heading
SPEx: Sports Injury Surveillance
ER: External Rotation
IR: Internal Rotation
ROM: Range of Movement
GIRD: Glenohumeral internal rotation deficit
F: Females
M: Males
n: Number of participants
NR: Not reported
*p*-value: Probability value
