# Supplementary material for: Can resistance training improve throwing performance in handball players? A Systematic review and meta-analysis
Source: BMC Sports Sci Med Rehabil. 2024 Apr 16;16:85. doi: 10.1186/s13102-024-00872-y (PMC11020874; doi:10.1186/s13102-024-00872-y)
Supplement: Supplementary file 1 — Supplementary Material 1. [file 13102_2024_872_MOESM1_ESM.docx]

Supplementary files

Μοre details regarding the search strategy (e.g initial search, after remove dublicates, after reading titles and abstracts, after reading all article, final studies) and the methodological quality score (Pedro scale) you can find to the following link

<https://drive.google.com/drive/folders/1IYJvCfSUaYvTrla2o5YKckX7-S93oB3D?usp=sharing>
